# Malaria incidence and assessment of entomological indices among resettled communities in Ethiopia: a longitudinal study

**DOI:** 10.1186/s12936-014-0532-z

**Published:** 2015-01-28

**Authors:** Teshome Degefa, Ahmed Zeynudin, Ameyu Godesso, Yohannes Haile Michael, Kasahun Eba, Endalew Zemene, Daniel Emana, Belay Birlie, Kora Tushune, Delenasaw Yewhalaw

**Affiliations:** Department of Medical Laboratory Sciences and Pathology, College of Public Health and Medical Sciences, Jimma University, Jimma, Ethiopia; Department of Sociology, College of Social Sciences, Jimma University, Jimma, Ethiopia; Department of Health Services Management, College of Public Health and Medical Sciences, Jimma University, Jimma, Ethiopia; Department of Environmental Health and Technology, College of Public Health and Medical Sciences, Jimma University, Jimma, Ethiopia; Department of Statistics, College of Natural Sciences, Jimma University, Jimma, Ethiopia

**Keywords:** Malaria, Incidence, Anopheles, Entomological indices, Resettlement, Ethiopia

## Abstract

**Background:**

Population resettlement has been considered among factors that may increase risk of malaria transmission. This study reports, the impact of resettlement on malaria incidence and entomological indices among communities resettled in suburbs of Jimma town, southwestern Ethiopia.

**Methods:**

A cohort of 604 study participants (302 resettlers and 302 non-resettlers) was monthly followed-up from September to November 2013 using active case detection. Moreover, longitudinal entomological study was conducted from June to November 2013. Anopheline mosquitoes were collected using CDC light traps and pyrethrum spray catches. Sporozoite ELISA was performed to determine *Plasmodium* infection rates.

**Results:**

Overall, 112 malaria cases were recorded during the three-month follow-up, of which 74.1% of the cases were from resettlement villages. *Plasmodium falciparum* incidence from resettlement and non-resettlement villages was 52.5 and 14.5/1,000 person-months at risk, respectively. Resettlement villages were three times at higher risk of *Plasmodium* infection (OR = 2.8, 95% CI: 1.22-6.48). *Anopheles gambiae s.l.* was the predominant (86.6%) of all the collected anopheline mosquito species. *Plasmodium* sporozoite rate in the resettlement and non-resettlement villages was 2.1 and 0.72%, respectively. *Plasmodium falciparum* entomological inoculation rate (EIR) for *An. gambiae s.l.* in the resettlement and non-resettlement villages was 13.1 and 0 infective bites/person/night, respectively. Both sporozoite rate and EIR were significantly higher in the resettlement villages (p < 0.05).

**Conclusion:**

Resettled communities were at higher risk of malaria infection as compared to non-resettled communities. Special attention should be given to malaria control interventions during resettlement programmes.

## Background

Malaria is a serious problem in Ethiopia, where about 68% of the population lives in malaria-risk areas. It is endemic in Ethiopia with differing intensity of transmission, except in the central highlands, which are malaria-free. In 2009, three million suspected malaria cases were recorded and nearly 2.3 million (77%) were tested. Besides the individual suffering caused by the disease, malaria poses a significant economic burden in Ethiopia [1,2].

Malaria showed a decline in Ethiopia over the last ten years as a result of high coverage of key malaria control interventions. This is attributable to the introduction of artemisinin-based combination therapy (ACT), use of rapid diagnostic tests (RDTs) at the peripheral health facilities, wide-scale distribution of long-lasting insecticidal nets (LLINs) and high coverage of sprayed households through targeted indoor residual spraying since 2004/2005 [3]. The National Malaria Indicator Survey (MIS) also showed that malaria declined in the country [4]. Shargie *et al.* [5] reported that malaria in Ethiopia decreased from 4.1% in 2006 to 0.4% in 2007 following scale-up of LLINs and large roll-out of ACT. Jima *et al.* [6] also reported that malaria inpatient admissions and deaths declined threefold between 2005 and 2009. Inpatient malaria cases and deaths in children less than 5 years of age reduced by 73% and 62%, respectively in Ethiopia [7]. However, the high influx of non-immune people into malaria endemic areas for social and economic reasons such as resettlement and search for alternative income, and the expansion of agricultural and other development projects could alter the number of malaria cases and malaria incidence.

In Ethiopia, information on the impact of resettlement on malaria incidence and transmission is scarce. However, few studies conducted in Pawe area, Northwest Ethiopia documented the emergence of malaria following resettlement schemes [8-10]. Currently, several urban development projects are underway in different parts of Ethiopia. In Jimma town, development projects such as road construction, airport expansion, housing, expansion of industrial zone and governmental institutions like schools and health facilities are being carried out. These development project activities resulted in resettlement of people from the centre to the suburbs of the town. The resettlement could result in ecological transformation, which in turn may affect malaria incidence and transmission dynamics. Therefore, the aim of this study was to investigate the impact of the resettlement on malaria incidence and transmission intensity in Jimma town, southwestern Ethiopia.

## Methods

### Study setting

**Figure 1:**
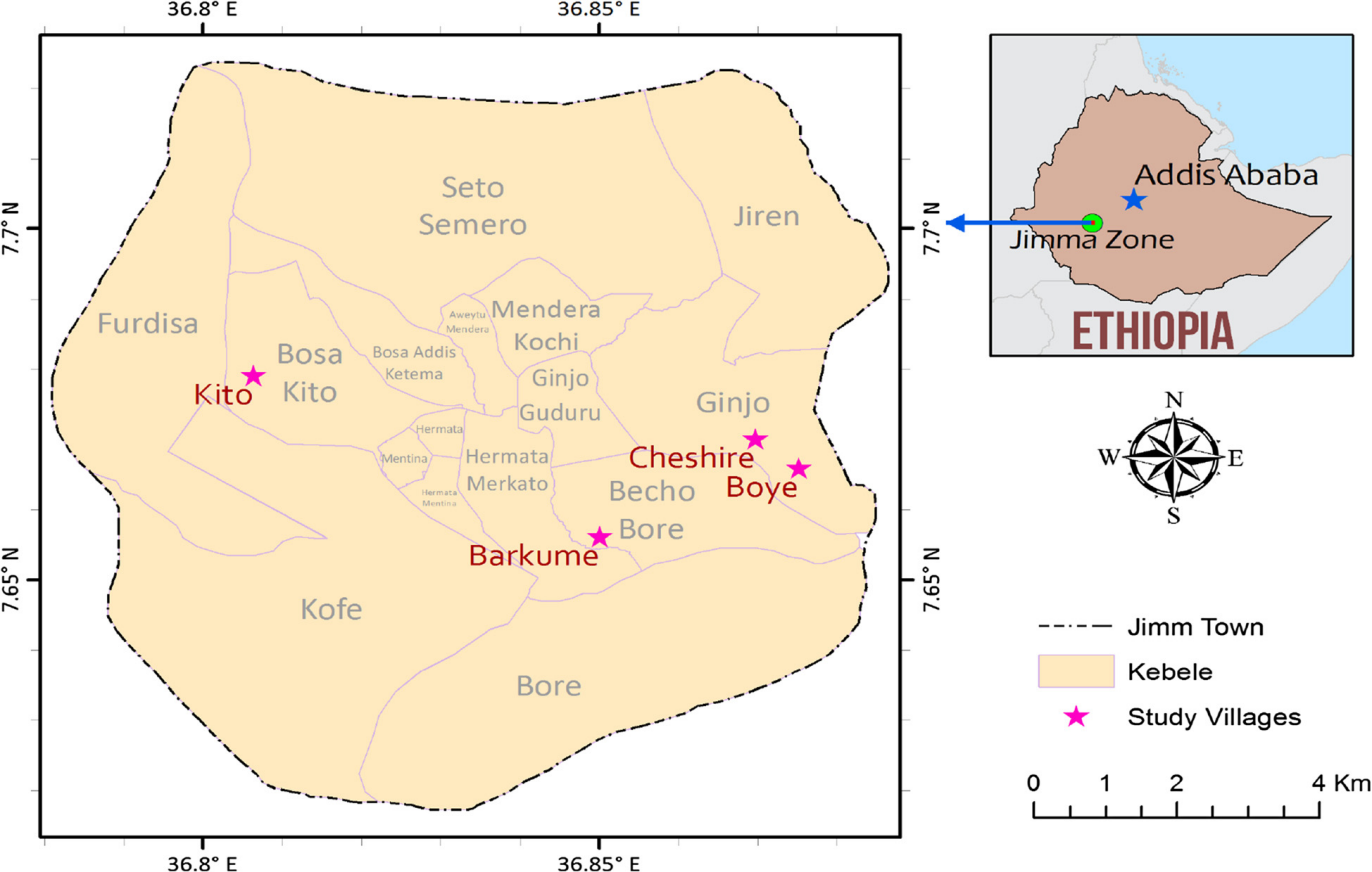
**Map of the study villages.**

The study was carried out in two resettlement and two control (non-resettlement) villages of Jimma town, southwest Ethiopia (Figure 1). Jimma town is located 350 km southwest of Addis Ababa. The geographical coordinates of the town are approximately 7°41′ N latitude and 36° 50′E longitude. The resettlement villages are Barkume and Kito while the control villages are Boye and Cheshire. All villages are suburbs of Jimma town. Communities resettled in the two resettlement villages in 2008. The resettlement was mainly due to airport expansion, road construction, expansion of school and health facility, industrialization and flooding problems at the centre of the town.

The control villages were selected purposefully based on the following criteria: i) Comparable socio-economic condition between resettlement and non-resettlement villages ii) Similarity in eco-topography between resettlement and non-resettlement villages, iii) Minimum distance of 3km between the two villages considering anopheline mosquito’s flight range, iv) Similarity in climate.

Jimma town is characterized by warm climate with a mean annual minimum and maximum temperature of 14°c and 30°c, respectively. The mean annual rainfall ranges from 1,138 to 1,690 mm. Maximum precipitation occurs during the three months (June to August), with minimum precipitation in December and January (National Meteorological Agency, Unpublished data).

### Study design and period

For parasitological survey, community-based cohort study was conducted using active case detection from September 1 to November 30, 2013. Moreover, longitudinal entomological study was conducted from June to November, 2013.

### Study participants and sampling

A cohort of 604 (302 from resettlement and 302 from non-resettlement villages) individuals residing in 202 households was followed-up. The sample size was determined using the formula for two population proportion with the following assumptions: Resettlement was considered as an exposure factor, previous malaria prevalence of 5.2% in Jimma town [11] was considered as an outcome in non-resettlement villages, 95% confidence level, 80% power and anticipated risk ratio of 2.96 [8,10]. Dropout rate of 10% and design effect of two (to adjust for clustering effect) were considered, which finally gave a sample size of 604 individuals.

Households were selected from each village by dividing the sample size to the average family size of the town [12]. Census of the resettlement villages was done prior to data collection and list of households of the non-resettlement villages was obtained from the *kebele* administration. Finally, simple random sampling was employed to select households from each village. All family members in the selected households fulfilling the inclusion criteria were enrolled in the study. Inclusion criteria of the study participants were: intention to remain in the study area for the duration of study follow-up, willingness to participate in the study and being permanent resident (in non-resettlement villages) and re-settler since 2008 (in resettlement villages). Individuals who were taking anti-malarial drugs at the baseline were excluded from the study.

### Socio-demographic data collection

Baseline data on socio-demographic and economic characteristics of each household member, use of malaria preventive measures and ownership of domestic animals were collected using semi-structured questionnaire. Presence and distance of temporary vector breeding sites from each household were recorded monthly.

### Parasitological survey

Parasitological data were collected by active case detection. A unique identification card was given to each household member. Each study participant was visited monthly for three months. During monthly house-to-house visit, blood sample was collected from the study participants following standard protocol [13]. Thick and thin blood films were prepared, stained with 10% Giemsa and examined at Medical Parasitology Laboratory in Jimma University. Experienced laboratory technologist examined the slides using oil immersion objective. All positive slides and 10% of the negative slides were re-examined by another blinded senior laboratory technologist.

### Entomological survey

Adult anopheline mosquitoes were collected from both resettlement and non-resettlement villages. For the indoor mosquito sampling, adult anopheline mosquitoes resting inside human habitations were collected monthly from 10 selected houses per village using pyrethrum spray catches (PSCs) and from two houses per village at fortnight interval using Centers for Disease Control and Prevention (CDC) light trap catches (LTCs) (John W. Hock Ltd, Gainesville, FL., USA).

Pyrethrum spray catches were conducted early in the morning from 06:00 to 08:00 hr. White sheets of cloth and Baygon aerosol (SC. Johnson & Son. Inc, USA) were used for the spray. After obtaining consent from the inhabitants, foodstuff and utensils were removed before spraying. Openings and eves which allow mosquito escape from houses were covered and white sheets were spread over the floor. Finally, rooms were sprayed with the aerosol for about 10 minutes. After 15 minutes following spraying, the sheet was removed and taken outside to inspect and collect the knockdown mosquitoes. CDC light trap was set indoor near the bed at a height of 1.5 meter from 18:00 to 06:00 hr in the selected houses to collect endophagic anopheline mosquitoes. For the outdoor mosquito sampling, CDC light trap was also set in the vicinity of sentinel houses from 18:00 to 06:00 hr.

The collected mosquitoes were transported to the Vector Biology Laboratory and sorted by genus and sex. The mosquitoes were morphologically identified to species using taxonomic keys [14,15]. Female anopheline mosquitoes were kept individually in eppendorf tube over silica gel for further processing.

### Sporozoite ELISA

Dried head and thorax of the preserved anopheline mosquito specimens were carefully separated from the abdomen and tested for *Plasmodium falciparum*, *Plasmodium vivax* 210 and *P. vivax* 247 circumsporozoite proteins (CSPs) simultaneously using ELISA [16,17].

### Data analysis

Data were coded, checked for completeness and entered into a computer. The data were then analysed using SPSS software package version 16.0 (SPSS, Chicago, IL, USA). At baseline, household and individual-level comparison was made in terms of socio-demographic and socio-economic characteristics between resettlement and non-resettlement villages using chi-square test. *Plasmodium falciparum* incidence rates were estimated as the number of cases per 1000 person-months at risk.

Generalized liner mixed effect model (GLMM) was used to compare the risk of *Plasmodium* infection between resettlement and non-resettlement villages. Let Y_ijk_ be the k^th^ binary outcome measure (malaria status) for individual (IND) j living in household (HH) i. The following three-level logistic mixed model was considered which accounted for the clustering of the outcomes within individuals and households.

$$ {Y}_{ijk}\sim Bernoulli\left({\pi}_{ijk}\right) logit\left({\pi}_{ijk}\right)= \log \left(\frac{\pi_{ijk}}{1-{\pi}_{ijk}}\right)={\beta}_0+{\beta}_1{x}_{i1}+\dots +{\beta}_k{x}_{ik}+{a}_i+{b}_{j(i)},{a}_i\sim N\left(0,{\delta}_{HH}^2\right),\ {b}_{j(i)}\sim N\left(0,{\delta}_{IND}^2\right) $$

The household effects *a*_i_represent the fact that some households are more successful in utilizing protective methods for malaria than others. The individual effects *b*_*j(i)*_ represent the fact that susceptibility to *Plasmodium* infection is not the same among individuals. Thus, comparison between resettlement and non-resettlement villages was adjusted for the possible confounding factors such as age, gender, distance from vector breeding site, insecticide-treated nets (ITNs), indoor residual spraying (IRS), presence of domestic animals in or around the houses, family size and type of house.

The sporozoite rate was estimated as the proportion of mosquitoes positive for *P. falciparum* and *P. vivax* of the total number of mosquitoes tested. Monthly *Plasmodium* EIRs of *Anopheles gambiae s.l.* was calculated from mosquito collections by LTCs using the standard formula, 1.605 × (no. circumsporozoite-positive ELISA results from CDC LTCs/no. mosquitoes tested) × (no. mosquitoes collected from CDC LTCs/no. catches) × No. days per month [18].

Analysis of variance was employed to compare resettlement and non-resettlement villages in terms *An. gambiae s.l.* density, sporozoite rate and EIRs. SPSS version 16.0 (SPSS, Chicago, IL, USA) and SAS version 9.3 (SAS, Cary, NC, USA) statistical software packages were used for the analysis. p < 0.05 was considered significant during the analysis.

### Ethical clearance

Ethical clearance was obtained from the Research and Ethics Review Committee of Jimma University. Permission was sought from Jimma Town Health Office. Written informed consent was obtained from heads of the households and parents/guardians for children. During parasitological survey, participants found positive were treated according to the national malaria diagnosis and treatment guideline.

## Results

### Socio-demographic characteristics of the study participants

Table 1 shows the socio-demographic and socio-economic characteristics of the study participants. A cohort of 604 study participants residing in 202 households was followed-up in the longitudinal parasitological study. Of the total households interviewed, 134 (66.3%) were females and 68 (33.7%) were males. The majority of the households (83.2%) were married and their mean age was 33.5 years. The mean family size was 4.49. There was no significant difference in socio-economic status of the households between resettlement and non-resettlement villages (p > 0.05). The majority (92.1%) of the houses were mud plastered. Most of the study households in the resettlement villages (62.4%) were located <1 km from mosquito breeding sites. The majority of households (63.4%) reported to have at least one ITN. Moreover, 86.1% of the households reported that their houses were sprayed with insecticides. There was significant difference in the type of house and distance of houses from mosquito breeding site between the resettlement and non-resettlement villages (p < 0.05).

**Table 1:** **Socio-demographic and socio-economic characteristics of heads of the households, Jimma town, Southwest Ethiopia**

**Variables**		**Village type**			**p-value**
		**Resettlement**	**Non-resettlement**	**Total**	
		**n (%)**	**n (%)**	**n (%)**	
Gender	Male	31 (45.6)	37 (54.4)	68 (100.0)	0.372
	Female	70 (52.2)	64 (47.8)	134 (100.0)	
Age group (years)	18-25	29 (43.9)	37 (56.1)	66 (100.0)	0.484
	26-30	23 (52.3)	21 (47.7)	44 (100.0)	
	>30	49 (53.3)	43 (46.7)	92 (100.0)	
Educational status	Illiterate	48 (55.2)	39 (44.8)	87 (100.0)	0.528
	Grade 1-4	19 (45.2)	23 (54.8)	42 (100.0)	
	Grade 5-8	27 (49.1)	28 (50.9)	55 (50.0)	
	Grade 9-10/11-12	7 (38.9)	11 (61.1)	18 (100.0)	
Main occupation	Private business	12 (54.5)	10 (45.5)	22 (100.0)	0.112
	Government employee	3 (33.3)	6 (66.7)	9 (100.0)	
	House wife	40 (47.6)	44 (52.4)	84 (100.0)	
	Daily laborer	38 (56.7)	29 (43.3)	67 (100.0)	
	Farmer	8 (57.1)	6 (42.9)	14 (100.0)	
	Others	0	6 (100.0)	6 (100.0)	
Estimated monthly income (ETB)	<500	20 (45.5)	24 (54.5)	44 (100.0)	0.266
	501-1000	57 (47.9)	62 (52.1)	119 (100.0)	
	>1000	24 (61.5)	15 (38.5)	39 (100.0)	
Distance from vector-breeding site	<1 km	75 (59.5)	51 (40.5)	126 (100.0)	0.000*
	>1 km	26 (34.2)	50 (65.8)	76 (100.0)	
House type	Mud plastered	89 (47.8)	97 (52.2)	186 (100.0)	0.037*
	Break wall	12 (75.0)	4 (25.0)	16 (100.0)	
ITN	Yes	60 (46.9)	68 (53.1)	128 (100.0)	0.243
	No	41 (55.4)	33 (44.6)	74 (100)	
IRS	Yes	86 (49.4)	88 (50.6)	174 (100.0)	0.684
	No	15 (53.6)	13 (46.4)	28 (100.0)	
Presence of domestic animal(s)	Yes	57 (55.3)	46 (44.7)	103 (100.0)	0.122
	No	44 (44.4)	55 (55.6)	99 (100.0)	

*Significant at p < 0.05; ETB = Ethiopian Birr.

The majority (57.1%) of the participants were females. Fifty seven percent of the study participants were above the age of 14 years followed by age groups 5–14 (30.5%) and < 5 years (12.5%) with mean age of 21 years. The difference in gender and age of the study participants between resettlement and non-resettlement villages was not significant (p > 0.05).

### Parasitological survey

All of the study participants completed the first and second round of parasitological survey while 22 (3.7%) were lost to follow-up during the third round survey. The overall prevalence of malaria was 112 (6.3%). *Plasmodium falciparum*, *P. vivax* and mixed infections accounted for 58 (51.8%), 52 (46.4%) and 2 (1.8%) of the total cases, respectively.

**Figure 2:**
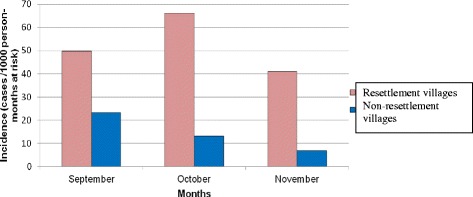
***Plasmodium falciparum*** **malaria incidence in resettlement and non-resettlement villages, Jimma town, Southwest Ethiopia (September – November 2013).**

Eighty three (74.1%) and 29 (25.9%) of the malaria cases were from the resettlement and non-resettlement villages, respectively. The majority of *P. falciparum* (77.6%) and *P. vivax* (69.2%) cases and two of the cases due to mixed infections were from resettlement villages. Figure 2 shows malaria incidence in the study villages. Overall, *P. falciparum* incidence was 33.5/1,000 person-months at risk, with 52.5/1,000 and 14.5/1,000 person-months at risk incidence in resettlement and non-resettlement villages, respectively.

Table 2 shows a generalized linear mixed regression model for comparison of *Plasmodium* infection between resettlement and non-resettlement villages. The difference in the risk of *Plasmodium* infection between resettlement and non-resettlement villages was significant (*x*^2^ = 27.64, p < 0.05). Individuals living in the resettlement villages were three times at higher risk of malaria infection (OR = 2.807, 95% CI: 1.22-6.48). House structure and family size were the main predictors of *Plasmodium* infection. The risk of *Plasmodium* infection among those who lived in houses with holes on the walls was three times higher as compared to those who lived in houses with no holes on the walls (OR = 3.18, 95% CI: 1.11-9.14). Family size also showed association with *Plasmodium* infection. For a decrease in family size by one, the odds of *Plasmodium* infection decreased by 24% (OR = 0.7589, 95% CI: 0.61-0.95). Other covariates assessed did not show significant effect at 5% level of significance.

**Table 2:** **Estimates of a three level generalized linear mixed regression model (GLMM) for comparison of malaria incidence between resettlement and non-resettlement villages, Jimma town, Southwest Ethiopia (September – November 2013)**

**Parameters**	**Exponentiated estimate (OR)**	**Standard error**	**95% CI**	**p-value**
Village type				
Non-resettlement (Ref.)	1.00			
Resettlement	2.807	0.427	1.22-6.48	0.016*
Month	1.210	0.179	0.85-1.72	0.286
Age	1.010	0.009	0.99-1.03	0.316
Gender				
Female (Ref.)	1.00			
Male	1.185	0.303	0.65-2.15	0.576
Distance of HH from larval breeding site				
Greater than 1 km (Ref.)	1.00			
Less than 1 km	2.250	0.432	0.97-5.25	0.060
Domestic animal				
No (Ref.)	1.00			
Yes	1.424	0.350	0.72-2.83	0.313
House type				
No holes on the walls (Ref.)	1.00			
Holes on the walls	3.186	0.537	1.11-9.14	0.031*
Family size	0.759	0.115	0.61-0.95	0.017*
ITNs				
Available (Ref.)	1.00			
Not available	1.250	0.335	0.65-2.41	0.506
IRS				
Sprayed (Ref.)	1.00			
Not sprayed	0.935	0.488	0.36-2.43	0.890

*Significant at p < 0.05;OR = odds ratio; 95% CI = 95% confidence interval.

### Adult mosquito abundance

Table 3 presents anopheline mosquito abundance, species composition and distribution. Overall, 1,912 female anopheline mosquitoes belonging to four species (*An. gambiae s.l., Anopheles coustani complex, Anopheles pharoensis* and *Anopheles squamosus*) were collected over the study period. *Anopheles gambiae s.l.* was the most predominant species (86.6%). The majority (76.3%) of anopheline mosquitoes were collected from the resettlement villages.

**Table 3:** **Anopheline mosquito species composition, abundance and distribution in resettlement and non-resettlement villages, Jimma town, Southwest Ethiopia (June – November 2013)**

**Village type**	**Anopheline species**	**Collection method**			
		**LTCs (Indoor)**	**LTCs (Outdoor)**	**PSCs**	**Total**
		**n (%)**	**n (%)**	**n (%)**	**n (%)**
Resettlement (n = 1458)	*An. gambiae* s.l	642 (44.0)	332 (22.8)	332 (22.8)	1306 (89.6)
	*An. coustani*	39 (2.7)	47 (3.2)	11 (0.8)	97 (6.7)
	*An. pharoensis*	29 (2.0)	12 (0.8)	6 (0.4)	47 (3.2)
	*An. squamosus*	1 (0.1)	7 (0.5)	0	8 (0.5)
Non-resettlement (n = 454)	*An. gambiae* s.l	146 (32.2)	84 (18.5)	119 (26.2)	349 (76.9)
	*An. coustani*	31 (6.8)	32 (7.0)	21 (4.6)	84 (18.5)
	*An. pharoensis*	3 (0.7)	1 (0.2)	7 (1.5)	11 (2.4)
	*An. squamosus*	4 (0.9)	6 (1.3)	0	10 (2.2)
Overall (n = 1912)	*An. gambiae* s.l	788 (41.2)	416 (21.8)	451 (23.6)	1655 (86.6)
	*An. coustani*	70 (3.7)	79 (4.1)	32 (1.7)	181 (9.5)
	*An. pharoensis*	32 (1.7)	13 (0.7)	13 (0.7)	58 (3.0)
	*An. squamosus*	5 (0.3)	13 (0.7)	0	18 (0.9)

Key: n = Number of anophelines collected; LTCs = Light trap catches; PSCs = Pyrethrum spray catches.

**Figure 3:**
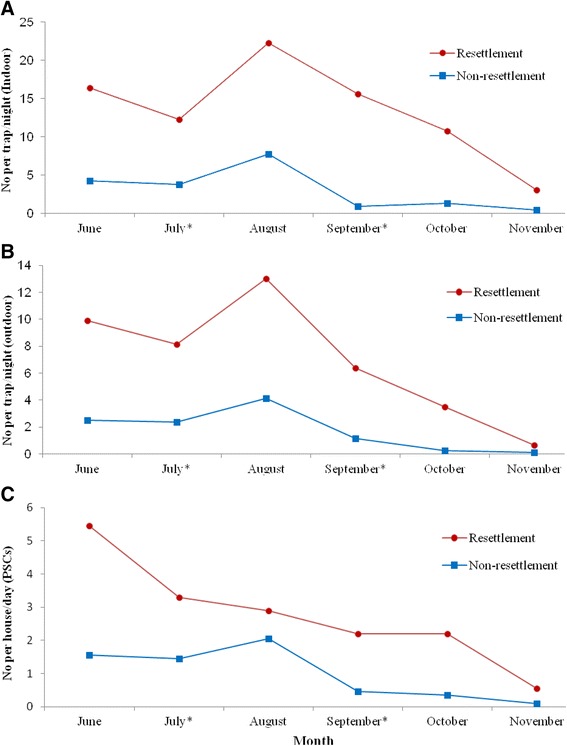
**Mean monthly indoor and outdoor** ***Anopheles gambiae s.l*** **density in resettlement and non-resettlement villages, Jimma town, Southwest Ethiopia (June – November 2013). (A)** Indoor host seeking mosquito density. **(B)** Outdoor host seeking mosquito density. **(C)** Indoor resting mosquito density.

Mean monthly indoor and outdoor density of *Anopheles gambiae s.l.* in the two villages is shown in Figure 3. Overall monthly density of indoor host seeking *An. gambiae s.l.* in the study area ranged from 1.96 to 15 per trap/night with mean density of 8.21 per trap/night. Mean indoor host seeking *An. gambiae s.l* density in the resettlement and non-resettlement villages was 13.38 and 3.04 per trap/night, respectively. The difference in mean density of host seeking *An. gambiae s.l* between the two villages was significant (F = 30.9, p = 0.0026). Mean indoor resting density of *An. gambiae s.l.* was significantly higher in resettlement villages (2.8 per house/day) than non-resettlement villages (0.99 per house/day) (F = 13.2, p = 0.015). Similarly, significantly higher mean outdoor host seeking density of *An. gambiae s.l.* was recorded in the resettlement villages (F = 18, p = 0.008).

### Sporozoite rates

A total of 1,510 anopheline mosquitoes were tested for *Plasmodium* CSPs. Of these, 23 *An. gambiae s.l.* specimens were positive for CSPs, of which 19 (82.6%) were positive for *P. falciparum* and 4 (17.4%) were positive for *P. vivax* (Table 4). In addition, a single specimen of *An. coustani* was also positive for *P. falciparum* (not PCR confirmed). Overall *Plasmodium* sporozoite rate for *An. gambiae s.l.* was 1.8%. The sporozoite rates of *P. falciparum* and *P. vivax* for *An. gambiae s.l.* were 1.5% and 0.3%, respectively. *Plasmodium* sporozoite rates for *An. gambiae* s.l. from indoor LTCs, outdoor LTCs and PSCs were 2.0%, 1.9% and 1.5%, respectively. Infectivity rates for *An. gambiae s.l*. in the resettlement and non-resettlement villages were 2.1% and 0.72%, respectively and the difference was significant (F = 9.4, p = 0.028).

**Table 4:** **Sporozoite infection rates of anopheles mosquitoes collected from resettlement and non-resettlement villages, Jimma town, Southwest Ethiopia (June–November 2013)**

**Collection method**	**Anopheline species**	**Village type**					
		**Resettlement**			**Non-resettlement**		
		**Number tested**	***Pf***	***Pv***	**Number tested**	***Pf***	***Pv***
			**n (%)**	**n (%)**		**n (%)**	**n (%)**
LTCs (Indoor)	*An. gambiae* s.l	450	9 (2.0)	2 (0.4)	102	0	0
	*An. coustani*	35	0	0	27	0	0
	*An. pharoensis*	27	0	0	3	0	0
	*An. squamosus*	1	0	0	4	0	0
LTCs (Outdoor)	*An. gambiae* s.l	248	4 (1.6)	0	75	1 (1.3)	1 (1.3)
	*An. coustani*	41	0	0	29	0	0
	*An. pharoensis*	12	0	0	1	0	0
	*An. squamosus*	7	0	0	6	0	0
PSCs	*An. gambiae* s.l	297	5 (1.7)	1 (0.3)	99	0	0
	*An. coustani*	13	1 (7.7)	0	19	0	0
	*An. pharoensis*	7	0	0	7	0	0
Overall	*An. gambiae* s.l	995	18 (1.8)	3 (0.3)	276	1 (0.36)	1 (0.36)
	*An. coustani*	89	1 (1.1)	0	75	0	0
	*An. pharoensis*	46	0	0	11	0	0
	*An. squamosus*	8	0	0	10	0	0

Key: *Pf* = *P. falciparum*, *Pv*210 = *P. vivax*; n = Number of anopheline mosquitoes positive for CSPs; Number in parenthesis indicate sporozoite rates.

### Entomological inoculation rates (EIRs)

Table 5 shows the entomological inoculation rates of *An. gambiae s.l.* in the study area. Overall the EIRs of *An. gambiae s.l.* for *P. falciparum* and *P. vivax* were 6.55 and 1.46 infective bites/person/month, respectively. The EIR of *An. gambiae s.l.* for *P. falciparum* in resettlement and non-resettlement villages was 13.1 and 0 infective bites/person/month, respectively, while EIR of *An. gambiae s.l.* for *P. vivax* EIRs in resettlement and non-resettlement villages was 2.9 and 0 infective bites/person/month, respectively. The difference in *P. falciparum* EIRs of *An. gambiae s.l.* between resettlement and non-resettlement villages was significant (F = 9.9, p = 0.025).

**Table 5:** **Monthly sporozoite infection rates and entomological inoculation rates (EIRs) of** ***An. gambiae*** **s.l. collected using LTCs from resettlement and non-resettlement villages, Jimma town, Southwest Ethiopia (June –November 2013)**

**Month**	**Village type**							
	**Resettlement**				**Non-resettlement**			
	***Pf*** **SR**	***Pf*** **EIR**	***Pv*** **SR**	***Pv*** **EIR**	***Pf*** **SR**	***Pf*** **EIR**	***Pv*** **SR**	***Pv*** **EIR**
June	2.4	19.0	0	0	0	0	0	0
July	1.3	7.9	1.3	7.9	0	0	0	0
August	0.7	8.2	0.7	8.2	0	0	0	0
September	4.1	30.5	0	0	0	0	0	0
October	3.2	17.0	0	0	0	0	0	0
November	0	0	0	0	0	0	0	0

Key: *Pf*SR = *P.falciparum* sporozoite rate; *Pf*EIR = *P. falciparum* entomological inoculation rate; *Pv*EIR = *P. vivax* entomological inoculation rate.

## Discussion

This study revealed that malaria incidence and transmission intensity was significantly higher in the resettlement villages as compared to the non-resettlement villages. Individuals living in resettlement villages were three times more likely to have *P. falciparum* infection as compared to individuals living in non-resettlement villages. The higher incidence of *P. falciparum* infection in resettlement villages might be due to suitable mosquito breeding sites created as a result of the resettlement [19]. Moreover, differences in housing structures in the two villages could also attribute for the observed difference in *P. falciparum* malaria incidence. The higher incidence of *P. falciparum* in the resettlement villages calls for malaria control intervention strategies during resettlement schemes.

Other studies have also reported increase in malaria incidence and an increase in malaria affected people as a result of population resettlement [20]. In studies conducted in Brazil, reemergence of malaria was noted as a result of resettlement [21,22]. Similarly, in another study conducted in Somalia [23], a significant increase in malaria prevalence was reported following influx of non-immune people in to malaria endemic areas. Earlier reports from Pawe resettlement scheme, North-west Ethiopia also showed similar findings [8,10,24]. Recently, Loha and Lindtjørn [25] also reported that temporary residents and visitors experienced higher episodes of *P. falciparum* compared to permanent residents which is in agreement with the findings of the current study.

The socio-demographic and socio-economic data indicated that the resettlement and non-resettlement study communities were similar with respect to age, sex, educational status, occupation of heads of the households, monthly income, family size, presence of domestic animals, ITN ownership and IRS. But, nearly three quarters of the households in the resettlement villages were located less than 1 km from vector-breeding sites. Moreover, 12% and 4% of the houses in the resettlement and non-resettlement villages were with holes on the walls, respectively. The overall malaria prevalence (6.3%) recorded in this study is slightly higher than the previous report from Jimma town, southwestern Ethiopia (5.22%) [11]. This could be due to the difference in time the two studies were conducted. The current study was conducted from August to October, which is part of the peak malaria transmission season in the study setting while the later was conducted from April to May. Moreover, environmental changes as a result of resettlement might have created potential breeding habitats for malaria vectors in the suburbs of the town. The present study was also conducted in suburbs of the town where resettlement occurred. Higher malaria incidence in peripheral areas than central areas of the same town was also reported in Burkina Faso [26].

In this study, the *P. falciparum* incidence (33.5/1,000 person-months at risk) is higher than malaria incidence of 3.6/10,000 person-weeks and 14.6 cases/1000 person-months at risk reported from Chano Mille [24] and Gilgel-Gibe hydropower area, southwestern Ethiopia [27], respectively. The difference could be due to differences in environmental factors and season. In contrast, *P. falciparum* incidence recorded in this study is lower than the incidence (90.5/1,000 population at risk) reported from Adama, central Ethiopia [28]. In the later, malaria incidence was determined from the total number of infections (*P. falciparum*, *P. vivax* and mixed infection, which might have contributed to the observed difference.

The entomological survey of this study showed that *An. gambiae s.l.* was the most predominant species in the study setting, accounting for 86.6% of all collected anopheline mosquitoes. Other similar studies also documented that *An. gambiae s.l.* is the most predominant species in Ethiopia [27,29,30].

Higher density of *An. gambiae s.l.* was recorded in the resettlement villages as compared to the non-resettlement villages. Indoor host-seeking density of *An. gambiae s.l.* was four times higher in resettlement villages. In addition, outdoor host-seeking density of *An. gambiae s.l.* was higher in the resettlement villages. These differences could be due to the presence of abundant temporary vector breeding sites in resettlement villages. Cox *et al*. [19] also reported that human settlement initially favors the multiplication of mosquito breeding sites and the perennial presence of high densities of mosquitoes as housing is quite often constructed faster than the drainage system. New settlers dug the land around their houses either for plastering or brick-making, resulting in pits which could create potential mosquito breeding sites hence increase vector density in areas with resettlement schemes [31]. Higher *P. falciparum* sporozoite rate in this study might have led to the occurrence of higher *P. falciparum* malaria cases than *P. vivax* in the study setting. Other studies also documented increased *Plasmodium* sporozoite rate with increased malaria incidence in southwestern Ethiopia [25,29].

*Plasmodium* sporozoite rate and mean monthly EIR of *P. falciparum* for *An. gambiae s.l.* were significantly higher in the resettlement villages. The higher EIRs observed in the resettlement villages indicate that malaria transmission intensity was higher in those villages. Heterogeneity in anopheline mosquito infectivity rate among villages was also reported in studies conducted in Ghana [32], Burkina Faso [25] and Sudanian area of Senegal [33]. Small-area variation in *Plasmodium* infectivity rates can be explained by the spatial heterogeneity of exposures to infection that is, human vector contacts.

The annual EIR of *An. gambiae s.l.* recorded in the present study is higher than the EIR reported from southern, central and south-central Ethiopia, with annual EIRs of 17.1, 34.8, 3.7 infective bites per year, respectively [29,34,35]. The difference might be due to difference in seasons of mosquito sampling. Anopheline mosquitoes were sampled during peak malaria transmission season in the present study unlike others which covered all seasons of the year. Moreover, difference in environmental factors between the study areas might have contributed to the difference in EIRs.

In addition, a single specimen of *An. coustani* was positive for *P. falciparum* CSP (not PCR confirmed). Although this species was not yet incriminated as vector of malaria in Ethiopia, it is a complementary vector elsewhere in Africa [36,37]. This species is considered as suspected vector in Ethiopia but never reported to have a role in malaria transmission.

## Conclusion

The study communities from resettlement villages were at higher risk of *Plasmodium* infection as compared to communities from non-resettlement villages. Malaria transmission intensity was also higher in the resettlement villages. Appropriate use of the existing malaria control intervention tools such as ITNs and IRS should be in place and scaled-up during resettlement schemes. Health education and environmental management supported by active community mobilization and participation is recommended to eliminate temporary and/or permanent vector-breeding sites in such new settlement areas. Further studies on the infectivity of *An. coustani* should be carried out using molecular diagnostics.
